# Validation of archived chemical shifts through atomic coordinates

**DOI:** 10.1002/prot.22756

**Published:** 2010-04-28

**Authors:** Wolfgang Rieping, Wim F Vranken

**Affiliations:** 1 Department of Biochemistry, University of CambridgeCambridge CB2 1GA, United Kingdom; 2 Protein Data Bank in Europe, European Bioinformatics Institute, Wellcome Trust Genome CampusHinxton, Cambridge CB10 1SD, United Kingdom

**Keywords:** nuclear magnetic resonance, chemical shift, protein, atom coordinates, validation

## Abstract

The public archives containing protein information in the form of NMR chemical shift data at the BioMagResBank (BMRB) and of 3D structure coordinates at the Protein Data Bank are continuously expanding. The quality of the data contained in these archives, however, varies. The main issue for chemical shift values is that they are determined relative to a reference frequency. When this reference frequency is set incorrectly, all related chemical shift values are systematically offset. Such wrongly referenced chemical shift values, as well as other problems such as chemical shift values that are assigned to the wrong atom, are not easily distinguished from correct values and effectively reduce the usefulness of the archive. We describe a new method to correct and validate protein chemical shift values in relation to their 3D structure coordinates. This method classifies atoms using two parameters: the per-atom solvent accessible surface area (as calculated from the coordinates) and the secondary structure of the parent amino acid. Through the use of Gaussian statistics based on a large database of 3220 BMRB entries, we obtain per-entry chemical shift corrections as well as *Z* scores for the individual chemical shift values. In addition, information on the error of the correction value itself is available, and the method can retain only dependable correction values. We provide an online resource with chemical shift, atom exposure, and secondary structure information for all relevant BMRB entries (http://www.ebi.ac.uk/pdbe/nmr/vasco) and hope this data will aid the development of new chemical shift-based methods in NMR. Proteins 2010. © 2010 Wiley-Liss, Inc.

## INTRODUCTION

Since the emergence of nuclear magnetic resonance (NMR) spectroscopy as a tool for determining molecular structure at the atomic level, it has contributed about 15% of all the protein and nucleic acid structures deposited at the Protein Data Bank (wwPDB).^1^,^2^ Parallel to the structural information, the related experimental NMR data can be deposited at the BioMagResBank (BMRB).^3^ This NMR data archive consists mostly of chemical shift values, an atom-specific NMR parameter that is highly sensitive to the local chemical environment,^4^ and contains a wealth of structural and dynamic information. Chemical shifts have an established role in determining protein secondary structure elements^5^–^7^ and backbone dihedral angles.^8^–^10^ More recently, chemical shift–based methods were developed to determine protein structure^11^–^13^ and flexibility.^14^,^15^ Many of these methods rely on the archived chemical shift information, sometimes in conjunction with the protein atom coordinate data. However, the archived chemical shift data are not always dependable, mainly because the chemical shift is a relative value that is calculated from an absolute frequency in relation to a reference frequency. This reference frequency should be based on standard referencing compounds and procedures.^16^–^20^ Despite the availability of these well-defined standards, alternative compounds are sometimes used (where the reference chemical shift is susceptible to sample conditions), the correct procedures are not followed, or other mistakes are made along the way.^8^,^17^,^20^–^23^

This large and important archive of chemical shift data is therefore not as reliable as it could be. Several methods have been developed that address this issue by correcting for the chemical shift dependence on nucleus (^1^H, ^13^C, and ^15^N) and atom type. The first database of corrected shifts was provided as part of the TALOS dihedral angle prediction protocol.^8^ That method is based on comparing the chemical shifts of backbone atoms in secondary structure elements to their expected value (as determined from the coordinate-derived (φ, ψ) surface) and applying a chemical shift correction where necessary. It only contains high-quality data. The RefDb database^24^ developed by the Wishart group uses the coordinate-based SHIFTX chemical shift prediction protocol^25^ and determines chemical shift corrections using the difference between SHIFTX-predicted and observed chemical shifts. This group also developed, as part of the PSSI program,^26^ a noncoordinate-based method based on secondary structure identification. Further coordinate-independent methods are LACS,^27^ which uses the difference between the chemical shift values of C^α^ and C^β^ atoms, and CheckShift,^28^ which compares the distribution of chemical shifts to a reference distribution based on the TALOS data.

The error rate in the archive is certainly reduced by use of these methods, but because the actual chemical shift corrections are not known for most archive entries, there is no absolute standard to compare to, and there can ultimately be no certainty about which method performs best. We think that several properties are desirable for any method that attempts to sanitize the chemical shift data: it has to provide a sound error estimate on the corrections it determines, it has to use as much information as possible to increase its robustness, and its mode of action has to be transparent.

Here, we present the Validation of Archived chemical Shifts through atomic COordinates (VASCO), a new correction method based on statistical analysis of a large set of chemical shift and coordinate data for all amino acid atoms.^29^ In this statistical study, we showed that the range of chemical shift values a given atom can adopt depends strongly on its solvent accessible surface area (ASA) as calculated from the atom coordinates: in short, atoms that are more exposed to solvent have narrower chemical shift distributions than atoms that are buried inside the core of a protein, and this holds true for side chain as well as backbone atoms. This dependency of the chemical shift of an atom on its ASA introduces a new dimension besides the well-known secondary structure effects, and we use this information in the VASCO approach to get better estimates of the chemical shift distribution available to a certain atom given its coordinates. The VASCO method thus uses side chain atom information, and further provides error estimates on the chemical shift correction per atom type as well as validating individual chemical shifts. The VASCO validated and corrected results are accessible from http://www.ebi.ac.uk/pdbe/nmr/vasco, and a full description of the file content is available as Supporting Information.

## MATERIALS AND METHODS

### Archived data

The preparation and analysis of archived data, and the generation of graphs, was described previously,^29^ except for the changes outlined in this paragraph. The per-atom solvent ASA is calculated using the WHATIF^30^ web service. WHATIF calculates ASA values in discrete values amounting to multiples of ∼0.43% of the in-vacuum surface of the atom in question. These discrete ASA values are directly used in VASCO. However, for the generation of graphs, the per-atom ASA values were perturbed to within 0.43% of their calculated ASA, so that the data points spread out along the *y* axis and thus give a better visual indication of their density (as opposed to a single vertical line containing all the data points for one discrete value). The 0.43% error introduced this way is much smaller than the expected error on the ASA itself, given the uncertainty of the calculation of atom coordinate positions and their inherent dynamic behavior in proteins.

The process of matching the BMRB protein sequence to the PDB sequence is based on the Needleman-Wunsch algorithm,^31^ which improves the linking of the chemical shift data to the atom coordinates by better exclusion of nonmatching residues and by treating gaps between the sequences correctly. Finally, the original data set was extended to a total of 3220 BMRB entries with 2781 unique matching PDB entries. The chemical shift corrections from previously published methods are extracted from the corrected values by comparing them to the original values (TALOS) or by extraction from reference files containing the correction factors by atom type (RefDb, LACS, and CheckShift).

### Probabilistic modeling

For each BMRB entry, we derive separate correction factors for each of the ^1^H, ^13^C, and ^15^N nuclei. For the carbons, we assume that we are not dealing with a single factor because different types of NMR spectra (with possibly different referencing) are recorded for this nucleus. Instead, we partition atoms that share similiar physicochemical properties into groups, each with an individual correction factor: (1) aliphatic carbons (C_ali_), (2) aromatic carbons (C_aro_), and (3) carbons with no protons bound (C_noH_). We thus end up with three different carbon correction factors.

VASCO derives the correction factors based on how well a chemical shift for each atom matches the expected chemical shift distribution. To this end, we assume that the distribution of an experimental shift δ of some atom type has the same principal shape as the corresponding distribution found in the database, except that it is shifted up- or downfield by a correction factor *c*_g_. The correction factor depends on the atom's associated group g ε {H, N, C_ali_, C_aro_, C_noH_}. Given the large size of the database, we assume that the majority of the entries is correctly referenced (estimates indicate that up to 20% of ^13^C and 30% of ^15^N chemical shifts could be incorrectly referenced^23^), and that the errors on the incorrectly referenced entries are broadly distributed and therefore do not significantly disturb the overall distribution. The database distribution itself is modeled for each atom type individually by a Gaussian with a certain mean and variance. Apart from being atom type dependent, chemical shift values also depend on the solvent accessibility *a* of an atom^29^ as well as on the secondary structure state *b* of the parent residue (as determined by STRIDE^32^: α-helix, 3_10_ helix, π-helix, β-strand, turn (any), and random coil). Hence, the reference distribution of an atom type should depend on an atom's solvent accessibility as well as it parent residue's secondary structural state. However, instead of modeling the reference distribution as an explicit function of *a* and *b*, we account for this dependency by binning the shifts with respect to their solvent accessibility, conditional on the secondary structure state, and atom type. In other words, for each class α, which is described by atom type, secondary structure state, and solvent accessibility bin, we derived an individual database distribution with mean *s*_α_ and inverse variance *k*_α_. Each of these bins contains 200 data points, except for the bin with *a* 0.0, which could hold more, and the bin with highest *a*, which may contain less. In this study, atoms with a given *a* and *b* that belong to a class for which there are fewer than 200 observations were excluded from the rereferencing calculations.

Given these assumptions, we have the following relationship between measured shift δ_*i*_ of atom *i* and the correction factor of its associated group, *c*_g(*i*)_:


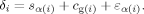
(1)

Here, *s*_α(*i*)_ denotes the average chemical shift of class ε_α(*i*)_ as found in the database and ε_α(*i*)_ a Gaussian error term with zero mean and an inverse variance equal to *k*_α(*i*)_. In probabilistic terms, we then arrive at the following probability for observing some shift δ_*i*_ given its class and correction factor:


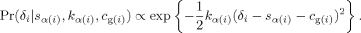
(2)

To infer the unknown correction factors from a data set of *n* measured shifts *D* = {δ_1_, …, δ_*n*_}, we use Bayes theorem.^33^ Assuming that the shifts δ_*i*_ are independent, the likelihood for observing the data *D* is a product of *n* individual distributions given in Eq. (2). After using the properties of the exponential function and some rearrangement of the exponent, we obtain the following posterior distribution for *c*_g_:


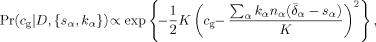
(3)

where we assumed a flat prior distribution for *c*_g_. Here, *n*_α_ and 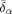, respectively, denote the number and average of the shifts in the data set that belongs to class α, and 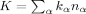. The posterior distribution captures the full information about possible values of the correction factors that can be derived from the experimental shifts given the model described above. To make numerical statements, we quantity the correction factors by their average


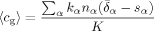
(4)

and uncertainty


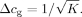
(5)

To ensure that incorrectly referenced entries have a minimal effect on the chemical shift distributions, the above procedure was first applied on the original data, the calculated correction factors were then applied on this data, and the procedure once again repeated. Further iterations of this procedure had no significant effect on the results.

Finally, we quantify the compatibility of a (corrected) individual shift δ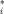 of a certain class with its reference distribution by calculating the *Z* score from the mean *s*_α(*i*)_ and variance *k*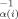 of the respective database distribution:


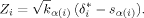
(6)

Generally, large *Z* scores indicate a discrepancy of a shift and the distribution found in the database, whereas compatible shifts lead to small *Z* scores.

## RESULTS

The chemical shift corrections determined by the VASCO method are compared to four published methods to investigate consistency between the results (Table I). The VASCO corrections correspond best to the TALOS data, except for the carbonyl atom where the corrections from the LACS and RefDb methods are more similar. The rms of the corrections for nitrogen backbone atoms vary widely and show that the results from the different methods are not consistent with each other. This variation is also evident from the error on the nitrogen atom corrections as determined by VASCO (Supporting Information). The difficulty in finding consistent corrections for these chemical shifts is likely caused by the dependence of the backbone nitrogen chemical shift on environmental factors like temperature and pH and illustrates the importance of determining the error on the chemical shift correction. Overall, the CheckShift corrections deviate the most from the VASCO ones. A possible reason for this is that CheckShift does not use the (informative) coordinate data, but relies on secondary structure prediction only, and might therefore give less reliable results overall. The range of correction deviations per atom between all methods was also determined, both for the maximal subset of entries between two methods and for the shared subset of entries for all methods (Table I). The VASCO deviations tend to be close to the lowest intermethod deviations, again with the exception of CheckShift. Although this seems to indicate that the VASCO corrections present some consensus over the TALOS, LACS, and RefDb values, it is impossible to determine which of these methods is “better,” as the actual chemical shift corrections are not known. However, there seems to be a consensus in the community that the TALOS data set is the most reliable, also because the corrected data are used for calibrating the TALOS dihedral angle prediction protocol and therefore have to be dependable. We therefore only present a more detailed comparison against the TALOS data.

**Table I:** Root-Mean-Square of the Difference Between the Chemical Shift Corrections from VASCO and Previously Published Methods

Atom name	Talos	LACS	RefDb	CheckShift	Intermethod (all)	Intermethod (shared)
N	0.57	n/a	0.67	0.63	0.70–0.79	0.66–0.79
H	0.07	n/a	0.13	n/a	0.09	0.09
H^α^	0.04	0.06	0.05	n/a	0.05–0.08	0.05–0.08
C^α^	0.15	0.19	0.21	0.55	0.19–0.52	0.18–0.45
C^β^	0.15	0.19	0.21	0.37	0.19–0.34	0.18–0.34
C	0.36	0.35	0.25	0.54	0.32–0.60	0.34–0.45

All values in ppm.

The range of the rms between the previously published methods is shown in the intermethod column for all possible combinations (all) and the subset of entries shared between the different methods for that atom (shared).

**Figure 1:**
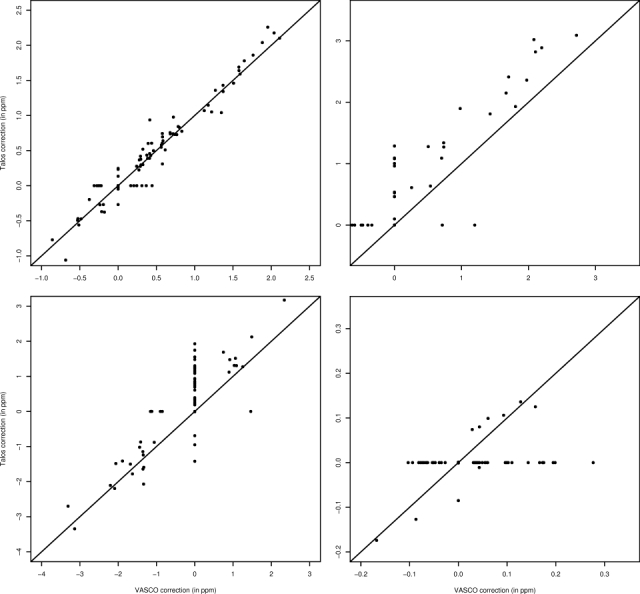
Chemical shift corrections from the TALOS database compared with the VASCO—calculated correction for C^α^ atoms (top left), C atoms (top right), N atoms (bottom left), and H^α^ atoms (bottom right).

In Figure 1, the chemical shift corrections reported in files from the TALOS database^8^ are compared to the corrections calculated by VASCO. The correspondence for the C^α^ atom corrections against the C_ali_ set of VASCO is excellent with a linear correlation of 0.978 (as determined by the Pearson method^34^). Note that a number of VASCO corrections are not present in the TALOS database. The match for the C atom corrections against the C_noH_ set of VASCO is not as good (0.885), with the VASCO correction consistently lower than the TALOS one (except for two values where VASCO determined a correction that was not present for TALOS). There is less data available for this set, which increases the error on the correction (Supporting Information). For amide nitrogens the correlation is 0.860. In this case, many TALOS corrections have a VASCO correction of zero. This happens because VASCO discards corrections that are smaller than three times their error: backbone N chemical shifts have a wide distribution, so the error on the correction VASCO determines is larger and this in turn makes many of these N chemical shift corrections unreliable (in total 2133 of 3100 corrections are discarded in this way). The TALOS corrections have no such error or reliability estimate. Finally, proton data are traditionally difficult to correct because the chemical shifts are very sensitive to the particular environment, and their variation is large in comparison to referencing errors. The few corrections that are available from the TALOS database, however, do correspond well with the VASCO corrections. VASCO is also able to reliably determine corrections for many other entries.

**Figure 2:**
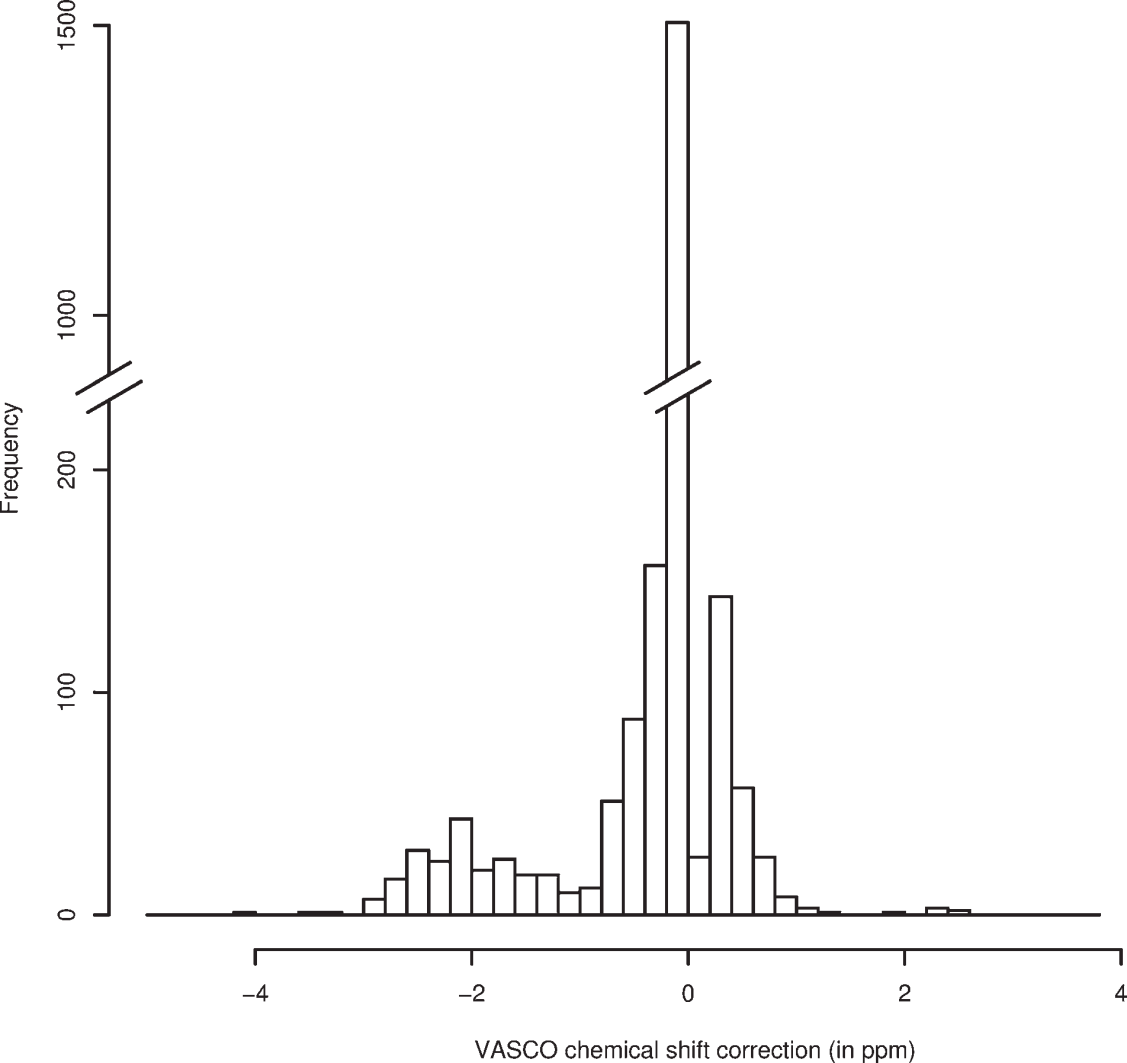
Histogram showing the distribution of the chemical shift corrections for aliphatic carbon atoms.

Further confirmation that the method determines relevant corrections is provided by the distribution of the corrections for the aliphatic carbons (Fig. 2). There is a cluster of correction values around 2 ppm. This corresponds to the difference between the carbon base frequency as set in Bruker spectrometers and the recommended carbon base frequency^19^ as calculated from the proton frequency with the standard γ ratio.

**Figure 3:**
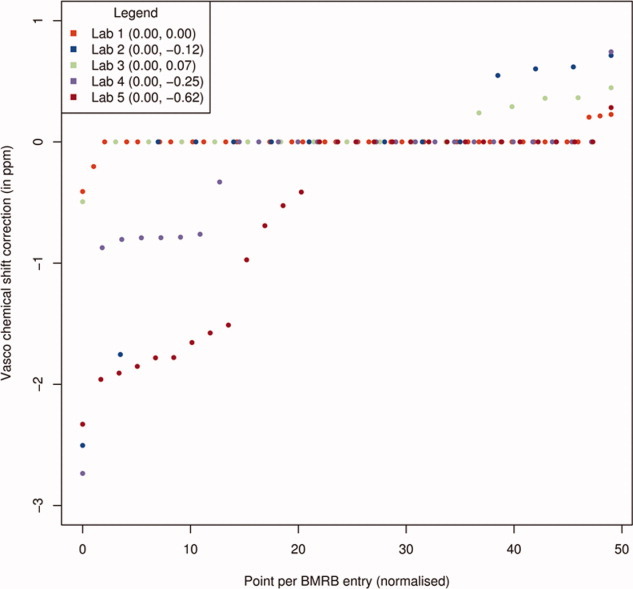
Chemical shift corrections for the aliphatic carbon atoms for selected NMR laboratories. The (median, average) corrections are listed behind the laboratory identifier.

Figure 3 shows the chemical shift corrections as determined for selected NMR laboratories. For a significant part of their submitted entries, some laboratories show a consistent negative correction (4 and 5), others a positive one (2 and 3). For comparison, only minor corrections were identified for Lab 1. VASCO can thus identify the use of different referencing procedures in some NMR laboratories.

The per-entry correction from VASCO is based on how well the chemical shift for each atom matches the expected chemical shift distribution. A Gaussian distribution is assumed, the mean and width of which are set based on the observed data, and a *Z* score is determined for each individual shift. Because the data are subdivided by per-atom ASA and secondary structure (as determined from the coordinates), chemical shift outliers can be identified more accurately by VASCO. For example, the expected chemical shift range of solvent-exposed atoms is smaller than for buried atoms in a secondary structure element.^29^ These *Z* scores are available in the online files (http://www.ebi.ac.uk/pdbe/nmr/vasco) and can be used to exclude outliers in an analysis if desired.

**Figure 4:**
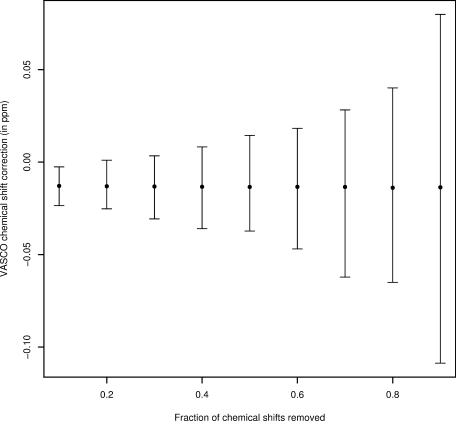
Variation of proton chemical shift correction for BMRB entry 7014 with increasing numbers of chemical shifts removed.

We also tested how stable the method is if the number of available chemical shifts is systematically decreased. For this purpose, we selected BMRB entry 7014, which has a large amount of chemical shift values (1228 chemical shifts for 116 residues) requiring no correction. The testing procedure randomly removed from 10 up to 90% of chemical shift values in steps of 10%. For each step, 10,000 samples were generated for which the chemical shift correction and its error were calculated. Although it is clear that the spread of correction factors increases as the number of chemical shifts decreases (Fig. 4 gives an example for protons), the error on the correction increases accordingly (data not shown). If the criterium is applied where corrections smaller than three times their error are removed, the correction is retained only in a very limited number of cases (see Table II). This shows that the method is very robust and is unlikely to suggest a correction unless supported by enough data.

**Table II:** Number of Samples (out of 10,000 for Each Step) Where a Valid Chemical Shift Correction Was Erroneously Found After Removing an Increasing Number of Chemical Shifts for BMRB Entry 7014

	Percentage of chemical shifts removed								
Atom class	10%	20%	30%	40%	50%	60%	70%	80%	90%
N	0	0	0	0	0	0	2	2	7
H	0	0	1	2	2	5	5	4	17
C_ali_	0	0	0	0	1	5	2	8	9
C_aro_	0	0	0	0	0	0	0	0	0
C_noH_	0	0	0	0	0	0	0	0	0

Finally, the graphs relating chemical shift values to the per-atom ASA as reported previously^29^ have been recalculated after applying the VASCO corrections and are available from http://www.ebi.ac.uk/pdbe/docs/NMR/shiftAnalysis/rereferenced.

## DISCUSSION

Although there is no “gold standard” with respect to chemical shift correction methods, the VASCO approach has several advantages: it is based on a very large statistical analysis of chemical shift information, uses coordinate data to increase the robustness and accuracy of the results, gives an error estimate of the chemical shift correction, and provides per-atom *Z* scores that can be used to flag chemical shift outliers. The method can be extended to use other information (e.g., dihedral angles) by further subdividing the data and to other nuclei and/or molecule types (e.g., RNA and DNA), although this only becomes possible with increasing data archive size. The future (and current) performance of VASCO is thus dependent on the size and coverage of the databases that underpin it. The decision by the wwPDB NMR task force to make deposition of chemical shifts mandatory at the PDB along with coordinates will have a great impact in this respect. We also note the potential of VASCO to become a useful tool for giving feedback to PDB depositors with regard to possible problems with chemical shifts (or coordinates).

The stability test on the VASCO method shows that it is very robust and is unlikely to suggest corrections even with decreasing numbers of chemical shift values. Because of the nature of the VASCO method and its dependence on a large body of statistical data, we could not devise other relevant internal tests of the method. After adding an offset to the chemical shifts, for example, VASCO will always directly return the exact offset value with the original error margin, while adding random scatter to the chemical shift values will only increase the error margin that VASCO calculates.

The extent of experimental data supporting VASCO is its main strength but also a source of potential problems, as both the incorporated chemical shift data and the coordinate data contain inaccuracies. However, because VASCO only uses subset distributions when a sufficient number of data points are available, we assume errors of this kind are lost in the overall satisfactory quality of the archive. We also use the corrected, not the original, chemical shift distributions to calculate the chemical shift corrections (see http://www.ebi.ac.uk/pdbe/docs/NMR/shiftAnalysis/comparison/exposure/html/ for examples on how the corrected chemical shift distributions compare to the original ones). Problems might occur for paramagnetic proteins, where large chemical shift deviations are present compared with diamagnetic proteins (which make up the major part of the database). Because of their unusual chemical shift values, these cases have a large error on the chemical shift correction and are not used.

The VASCO-corrected data archive already serves as the reference resource for a new method to predict random coil chemical shift values based on protein sequence and was instrumental in greatly increasing its prediction accuracy.^35^ We hope that the VASCO archive will help in improving other implementations that use chemical shift information and are committed to provide, on request, customized subsets of the data to address particular research questions.
