# Supplementary material for: Validation of archived chemical shifts through atomic coordinates
Source: Proteins. 2010 Apr 28;78(11):2482–9. doi: 10.1002/prot.22756 (PMC2970900; doi:10.1002/prot.22756)
Supplement: Supplementary file 1 [file prot0078-2482-SD1.pdf]

1 Graphs showing errors on the VASCO chemical shift correction.

These figures describe the VASCO errors on the chemical shift corrections in function of these chemical shift corrections. The red lines indicate the cutoff of 3 times the error - values on the left and right of these lines are valid VASCO corrections, values within the lines are not. Note that for nitrogen the error is often similar to the actual correction, and very few corrections can be used.

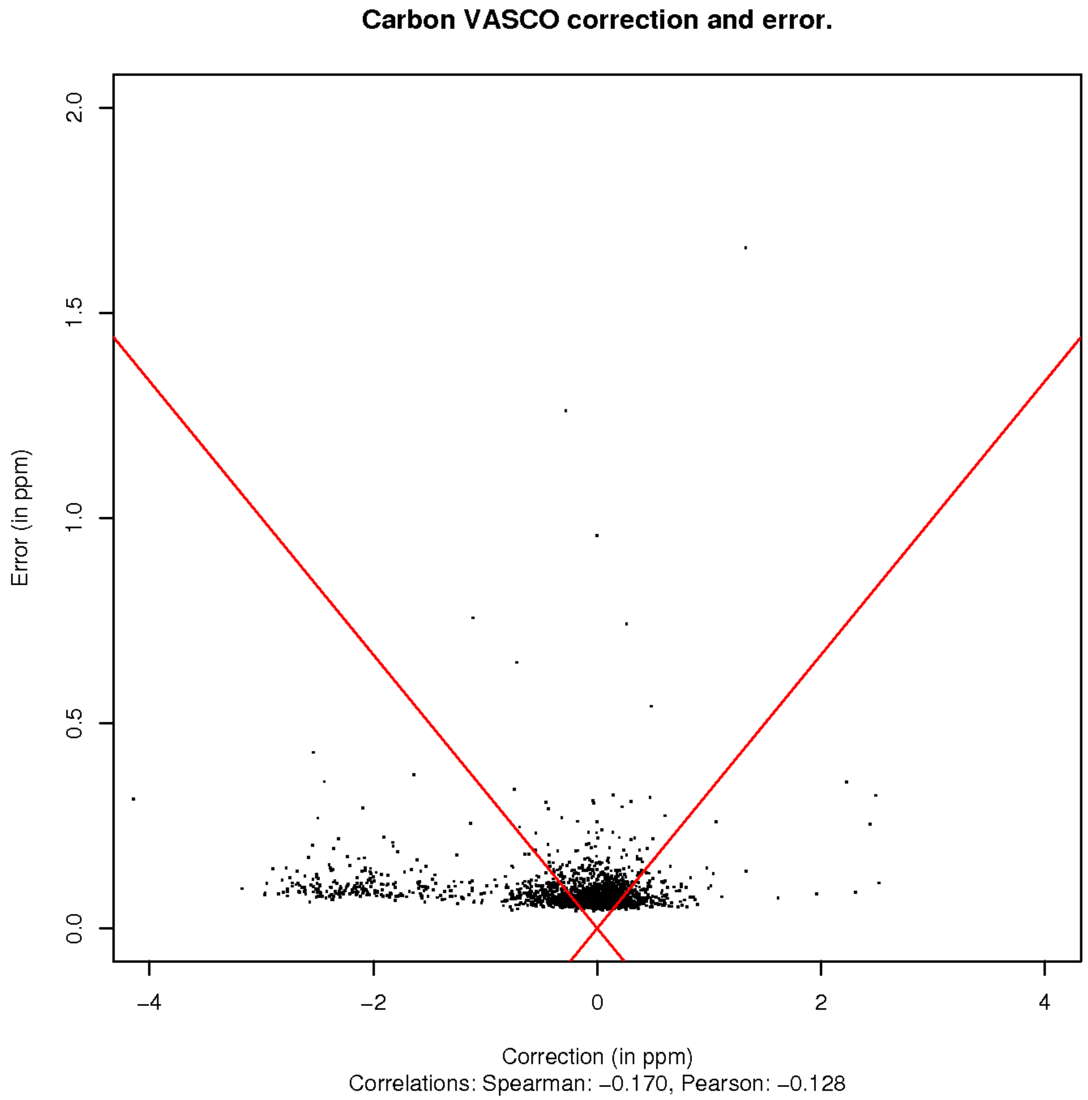

Figure 1: VASCO correction and corresponding error for the C_{ali} set for all entries.

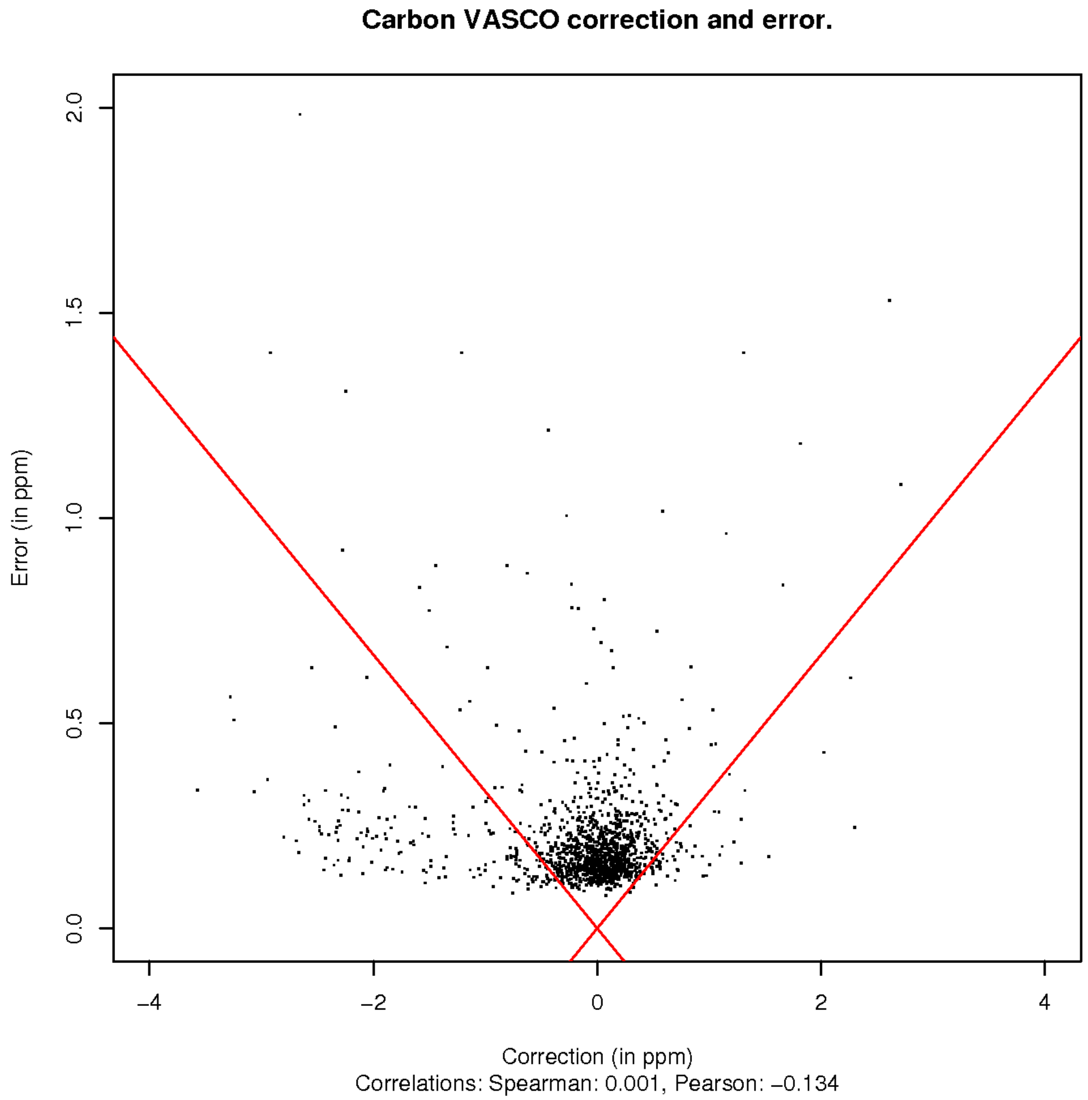

Figure 2: VASCO correction and corresponding error for the C_{aro} set for all entries.

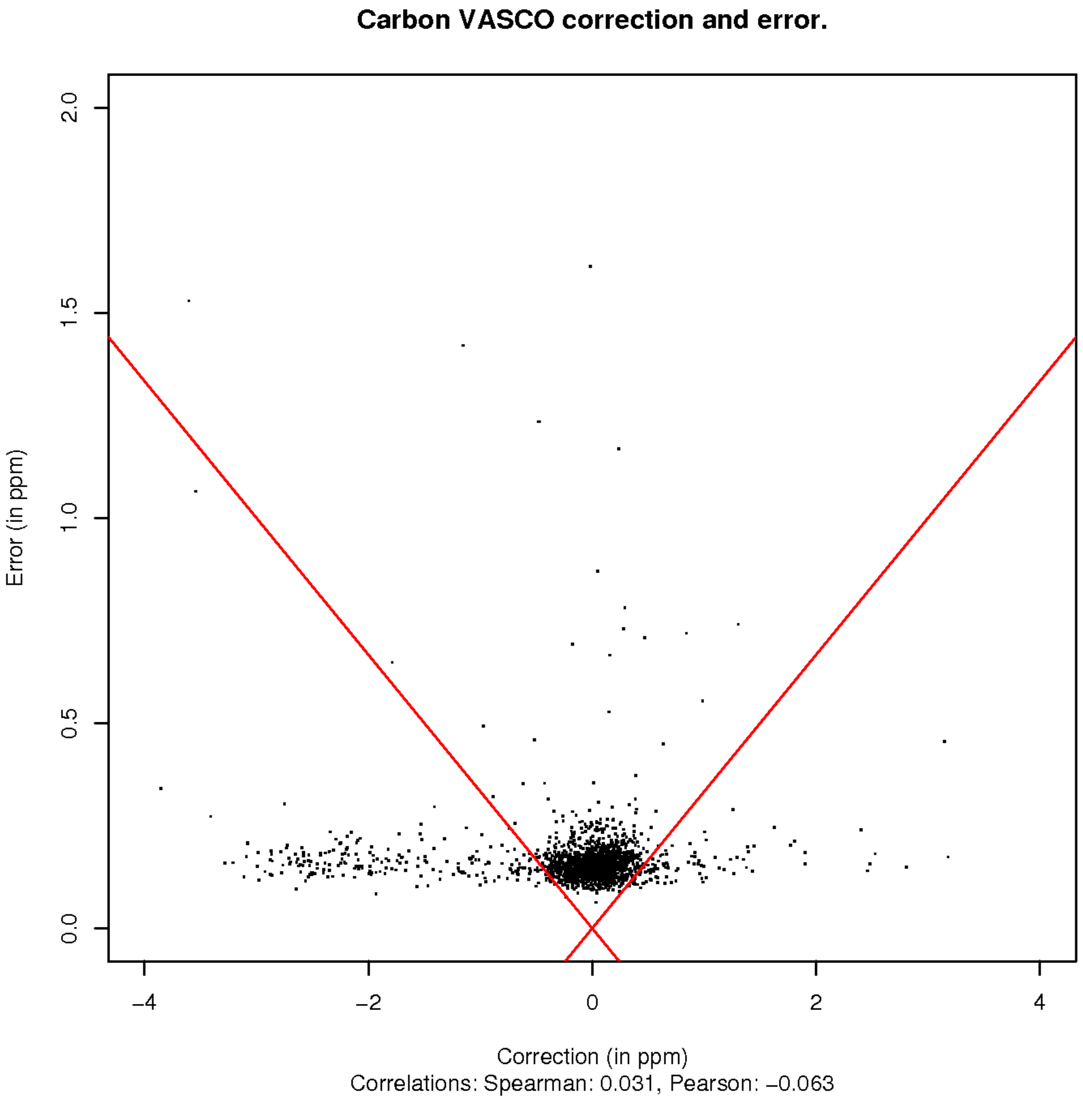

Figure 3: VASCO correction and corresponding error for the C_{noH} set for all entries.

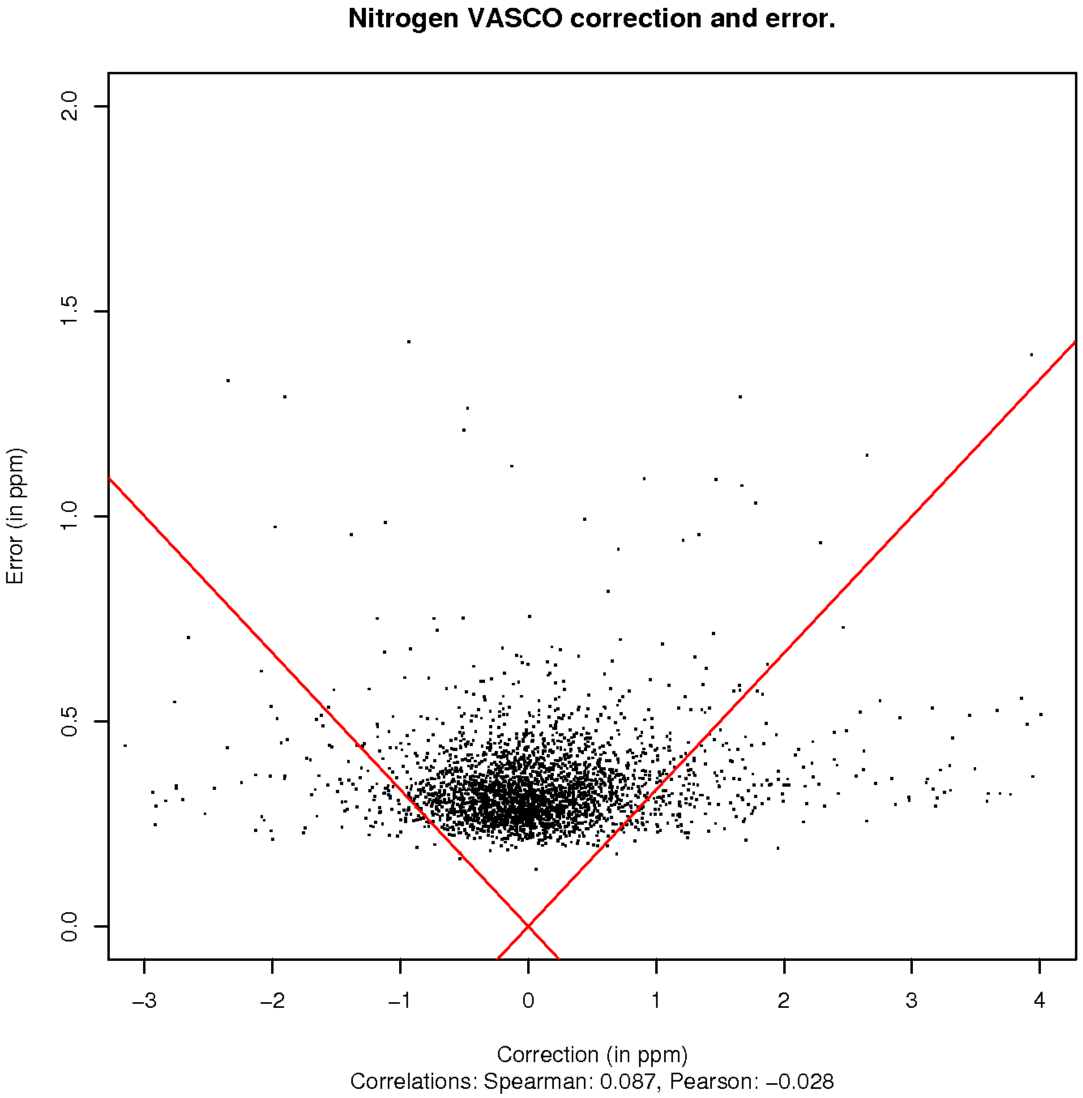

Figure 4: VASCO correction and corresponding error for the N (nitrogen) set for all entries.

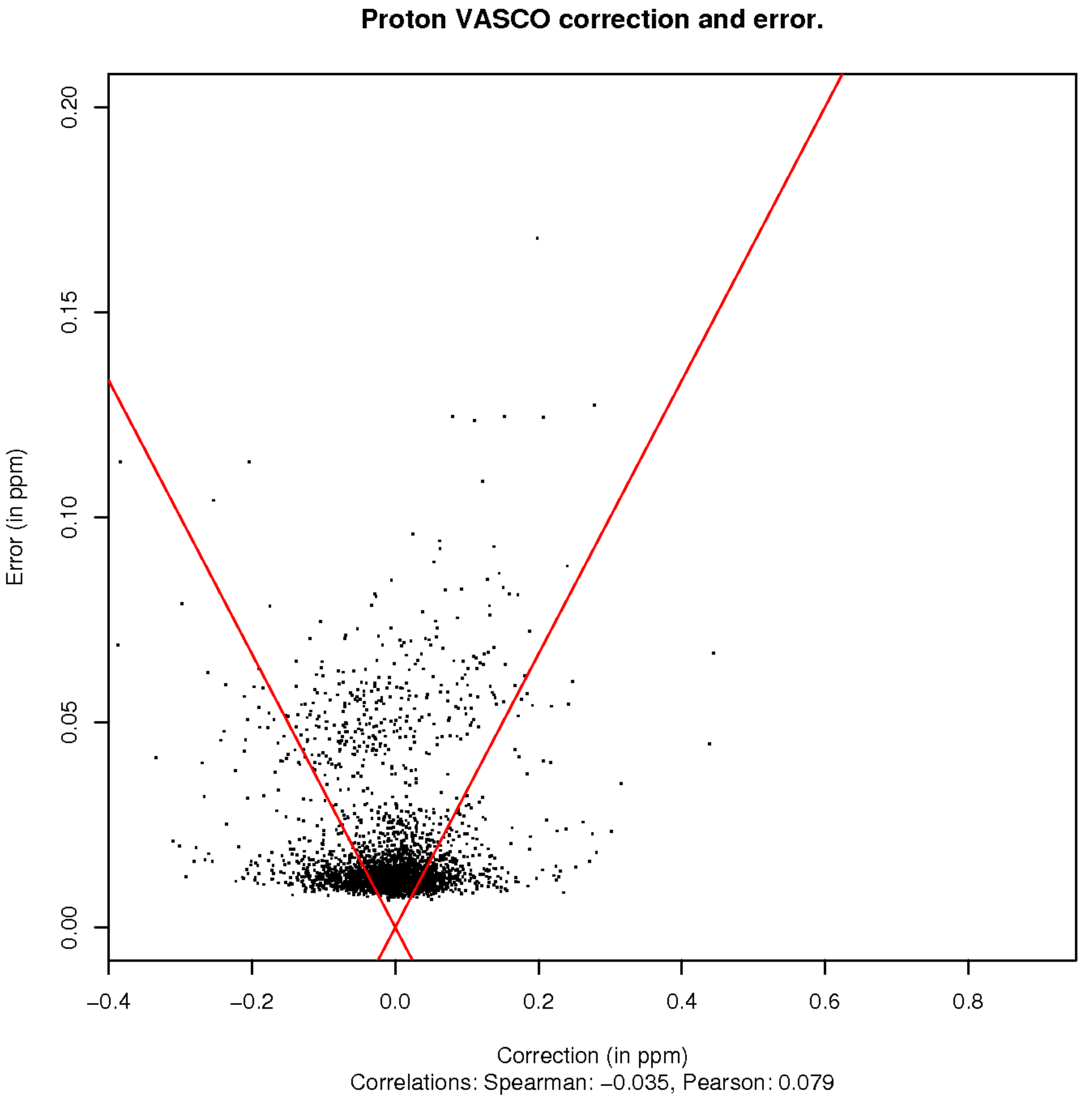

Figure 5: VASCO correction and corresponding error for the H (proton) set for all entries.
