# Supplementary material for: Validation of archived chemical shifts through atomic coordinates
Source: Proteins. 2010 Apr 28;78(11):2482–9. doi: 10.1002/prot.22756 (PMC2970900; doi:10.1002/prot.22756)
Supplement: Supplementary file 2 [file prot0078-2482-SD2.pdf]

VAlidation of Archived chemical Shifts through atomic Coordinates (VASCO) files

This document describes in detail the content of VASCO files. These files can be downloaded from http://www.ebi.ac.uk/pdbe/nmr/vasco/. The header of the VASCO files contains several content blocks, which are described in turn.

1. Metadata

```
#
# VASCO chemical shift information, corrected and validated using coordinate data.
#
# DATE              Generated on 2010-03-10, original BMRB deposition date 2001-03-07.
# BMRB ORIGIN       bmr9999
# PDB ORIGIN        1abc
# PDB EXPTYPE       NMR
#
```

This section contains information on:

- The date when the file was generated
- The date when the chemical shift data was deposited at the BMRB
- The BMRB ID from where the chemical shifts originate
- The code of the PDB entry that was selected to provide the coordinate information
- The experimental type of the PDB entry (NMR or X-RAY).

2. VASCO chemical shift correction.

```
#
# CORRECTION    C (aliphatic)                      1.569 +/- 0.079   APPLIED
# CORRECTION    C (high ppm, proton attached)     -0.000 +/- 0.000   NOT CALCULATED - NOT ENOUGH DATA
# CORRECTION    C (high ppm, no proton)           -0.000 +/- 0.000   NOT CALCULATED - NOT ENOUGH DATA
# CORRECTION    N                                  0.317 +/- 0.284   NOT APPLIED - UNCERTAIN
# CORRECTION    H                                 -0.000 +/- 0.010   ORIGINAL CORRECT
#
```

This section lists the chemical shift corrections calculated by VASCO. These corrections are calculated for five different atom classes:

1. **C (aliphatic)**: Aliphatic carbon atoms (*e.g.* an alanine C^{β}, a lysine C^{γ}).

2. **C (high ppm, proton attached)**: Carbon atoms with a proton attached that have high chemical shift values (*e.g.* the C^{δ} atoms of a phenylalanine).
3. **C (high ppm, no proton)**: Carbon atoms without an attached proton that have high chemical shift values (*e.g.* the backbone carbonyl atom).
4. **N**: Nitrogen atoms (all).
5. **H**: Hydrogen atoms (all).

Each atom class is followed by the chemical shift correction and the error on it as calculated by VASCO. Finally, a short comment describes what this correction means for this entry. Several values are possible:

1. **ORIGINAL CORRECT**: The original chemical shift values are correct according to VASCO.
2. **APPLIED**: A valid chemical shift correction was found and was applied to the chemical shift values found in this file. This happens when the chemical shift correction is less than 3 times the error on it.
3. **NOT APPLIED - UNCERTAIN**: A chemical shift correction was found, but was **not** applied to the chemical shift values found in this file. This happens when the chemical shift correction is uncertain (more than 3 times the error on it).
4. **NOT CALCULATED – NOT ENOUGH DATA**: Not enough chemical shift values are present for this atom class, and no correction or error on it could be calculated.

3. Match between protein sequence in BMRB and PDB file.

```
#
# SEQUENCE PDB   A MISSINGTHISISSOMESEQUENCE___
# BMRB MATCH       _______======DIFF========ADD
#
```

This section lists the match between the protein sequence in the BMRB and PDB files. For each chain from the PDB file (**SEQUENCE PDB**), the original chain code is listed (in this case **A**), followed by the sequence. The matching BMRB sequence (**BMRB MATCH**) is listed underneath. The following symbols are used in the comparison:

- An underscore (_) means that this residue is present in one sequence but not the other.
- An equal sign (**=**) means that this residue is the same in both sequences.
- A one-letter amino acid code in the BMRB MATCH sequence means that this residue is substituted compared to the PDB sequence.

4. Notes.

```
#
# Notes:
#
#  - The PDB sequence is used for information below
#
#  - The columns hold the following values:
#
#       1  Line_ID
#       2  Chain_code
#       3  Residue_code
#       4  Residue_label
#       5  Residue_stride_secondary_structure
#       6  Atom_name
#       7  Atom_accessible_surface_area     (for protons the heavy atom value is used)
#       8  Atom_type
#       9  Chemical_shift_value
#       10 Chemical_shift_Z_score           (missing values mean not enough statistical data
available)
#       11 Chemical_shift_ambiguity_code    (1 for unambiguous/stereospecific, 2 for not
stereospecifically assigned)
#       12 Comment
#
```

This section contains information on the columns in the chemical shift section.

1. **Line_ID**: A unique identifier for the data row in the file.

2. **Chain_code**: The chain code from the PDB file.

3. **Residue_code**: The residue sequence code from the PDB file.

4. **Residue_label**: The residue amino acid label (*e.g.* ALA for alanine, ...).

5. **Residue_stride_secondary_structure**: The secondary structure as determined by STRIDE. Values are:

   H: α helix.
   G: 3^{10} helix.
   I: π helix.
   E: Extended (β strand).
   T: Turn (any).
   C: Random coil.

6. **Atom_name**: The name of the atom (IUPAC).

7. **Atom_accessible_surface_area**: The per-atom accessible surface area as calculated by WHATIF. For protons the heavy atom value is listed.

8. **Atom_type**: The atom element.

9. **Chemical_shift_value**: The chemical shift value from the corresponding BMRB file.

10. **Chemical_shift_Z_score**: The Z-score of the chemical shift as calculated by VASCO for this atom in this secondary structure and with this per-atom accessible surface area. Following is a short description of illustrative values for the Z-scores for a chemical shift of an atom:

0.000	Exactly the statistically expected value.
1.000	This chemical shift value is exactly one standard deviation higher than the statistically expected value.
-2.533	This chemical shift value is 2.533 standard deviations lower than the statistically expected value.

11. **Chemical_shift_ambiguity_code**: As taken from the original BMRB file, and only applied when relevant for prochiral methylene atoms. A value of 1 means and unambiguous assignment (and stereospecific in case of prochiral methylenes), a value of 2 means that this prochiral methylene atom was not stereospecifically assigned.

12. **Comment**: A short text comment about this data line. Values are:

    a. **Shift outlier**: Marks a chemical shift value that is 3 standard deviations removed from the statistically expected value.

    b. **Z score missing**: No Z-score could be calculated for this chemical shift because insufficient information is available to calculate a good statistical distribution. Reasons are:

        i. **not enough data for atom**: There is not enough chemical shift data available for this atom with this per-atom accessible surface area as part of a residue with this secondary structure.

        ii. **not enough data for secondary structure**: There is not enough chemical shift data available for this atom as part of a residue with this secondary structure.

5. Data values.

```
1  A    2  GLU  C   H       6.220  H     8.450    0.220  1
2  A    2  GLU  C   N       6.220  N   125.400    1.238  1
3  A    2  GLU  C   HA      5.247  H     4.340    0.434  1
4  A    2  GLU  C   HB2    19.330  H     2.060    0.651  2
```

This section lists data values with the columns as listed in section 4.
